# Ultrasound and nerve stimulator guidance lumbar transforaminal epidural block for the treatment of patients with lumbosacral radicular pain

**DOI:** 10.1038/s41598-022-10021-5

**Published:** 2022-04-08

**Authors:** Seyed Ali Emami, Mehdi Sanatkar, Ebrahim Espahbodi, Seyed Khalil Pestehei

**Affiliations:** 1 Pain Research Center, Neuroscience Institute, Tehran University of Medical Sciences, Tehran, Iran; 2 Department of Anesthesiology and Critical Care, Imam Khomeini Medical Center, Tehran University of Medical Sciences, Tehran, Iran

**Keywords:** Medical research, Neurology

## Abstract

Transforaminal epidural block (TEB) is a widely accepted technique and minimally invasive procedure for the treatment of lumbosacral radicular pain. This study aimed to evaluate the accuracy, efficacy, and safety of ultrasound and nerve stimulator guidance lumbar transforaminal epidural block (UNTEB) for the patients with unilateral lower lumbar radicular pain. The accuracy of this method was evaluated by fluoroscopy. Using UNTEB via axial and the in-plane approach technique was performed in 42 segments of 25 patients who presented with lumbosacral radicular pain to lower extremities. The contrast medium was injected to evaluate the needle tip at the intervertebral foramen under fluoroscopic guidance. The numerical rating scale was used to assess pain before and after treatment. The success ratio of UNTEB in L3/L4 level was 100%, in L4/L5 was 95.4% and in L5/S1 was 100%. The numerical rating scale (NRS) for lumbosacral radicular pain improved from a mean from 7.8 to 2.8 1 day after procedure (p = 0.01) and from a mean from 7.8 to 2.4 1 week after UNTEB (p = 0.01). None of our subjects experienced any complications during and after the procedure. UNTEB with fluoroscopic validation is an accurate, effective, and safe method for short-term pain relief of the patients with unilateral lumbosacral radicular pain.

## Introduction

Inflammation or irritation of a spinal nerve root due to degeneration of the intervertebral disc induces unilateral radicular pain^1^. Treatment options of lumbosacral radicular pain range from conservative modalities such as medications and physiotherapy to surgical interventions^2^. TEB is a widely accepted technique and minimally invasive procedure for the treatment of lumbosacral radicular pain^3^. To visualization of the needle tip and navigation to the target point during the TEB, this procedure is performed under the fluoroscopy or computed tomography (CT)^4^. Many writers have sought to explain US-guided TEBbecause of the benefits of US-guided nerve block, and they have had a lot of success in the treatment of lumbar radicular pain^5,6^. Based on previous studies, fluoroscopy is the most reliable method for appropriate positioning of the needle during the TEB, but this manner cannot stablish the exact relation with clinical symptoms^6^. Electrical stimulation of the nerve root, especially a motor response at low amplitude, can cause symptoms of radicular pain during US-guided TEB; therefore, identify the proper level involved with radicular pain^7^. We use a nerve stimulator to improve the success of needle trajectory and correct target position during US-guided TEB. In the review of previous literatures on US-guided lumbar TEB, one study used both ultrasound and nerve stimulator guidance (double guide) during this procedure^8^. This study aims to evaluate the safety, accuracy, and pain relief of ultrasound and nerve stimulator guidance lumbar transforaminal epidural block (UNTEB) with fluoroscopic validation for short-term pain relief of patients with lumbosacral radicular pain.

## Materials and methods

**Figure 1:**
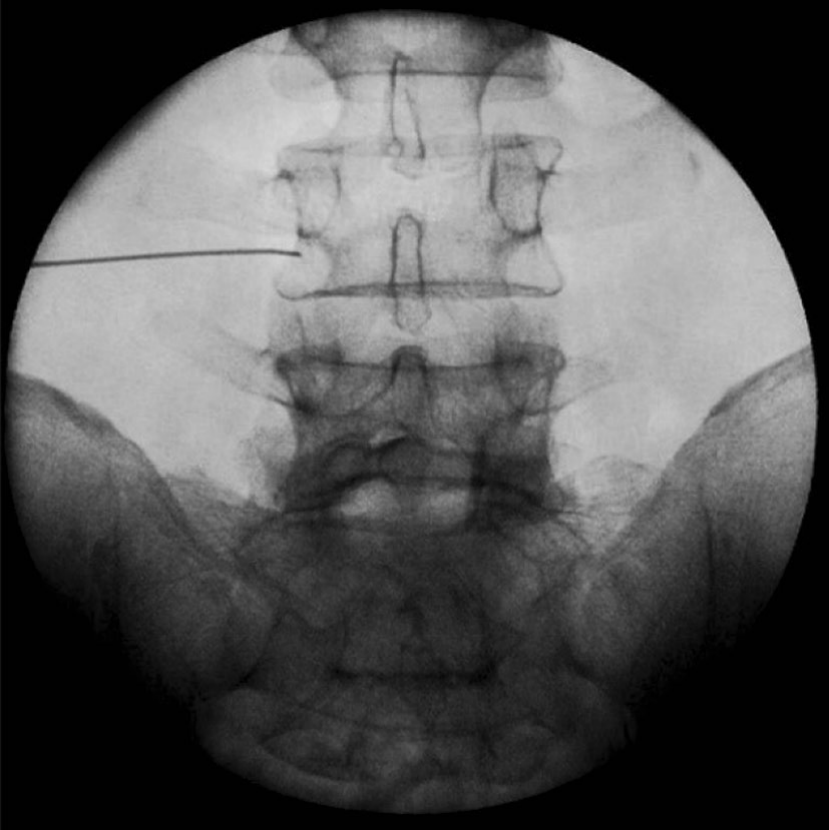
Fluoroscope verification of needle placement at L4/L5 in anterior–posterior view.

**Figure 2:**
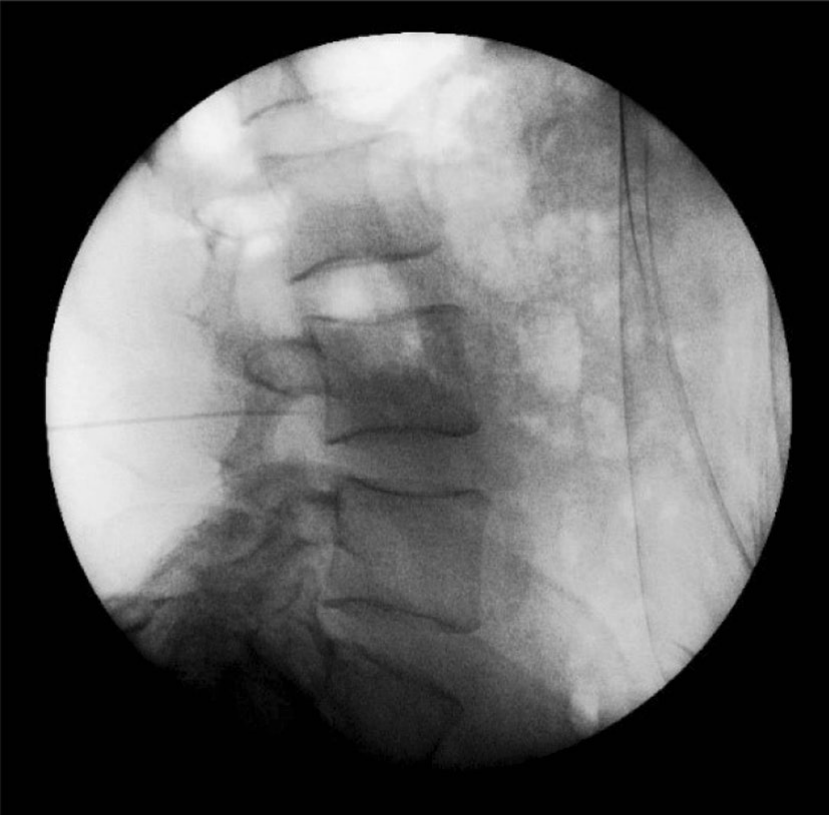
Fluoroscope verification of needle placement at L4/L5 in lateral view.

**Figure 3:**
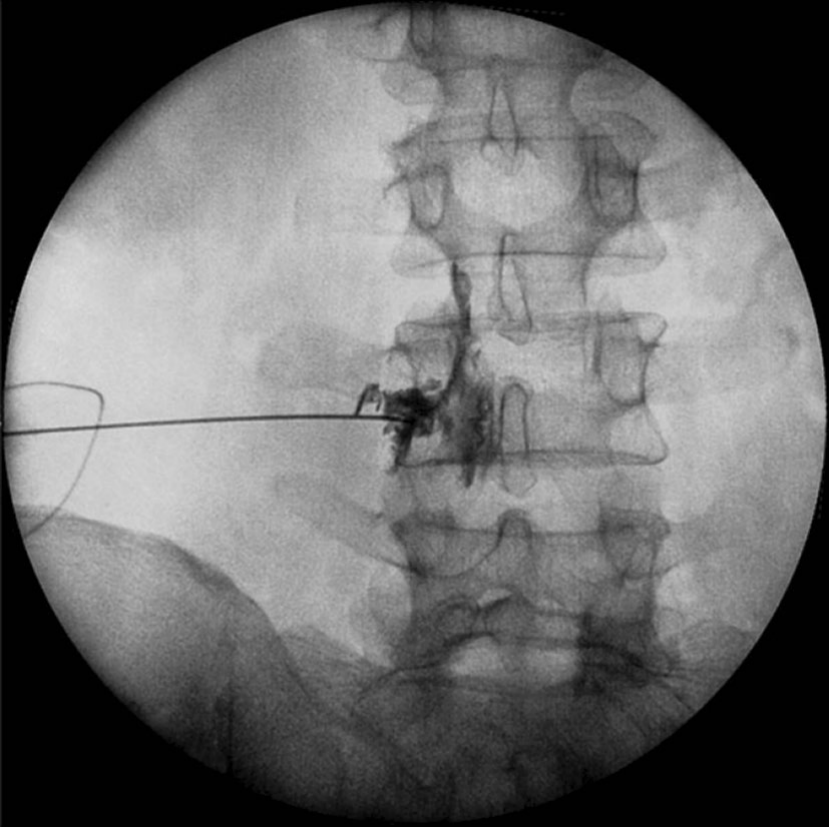
Fluoroscope verification of contrast spread at L4/L5.

**Figure 4:**
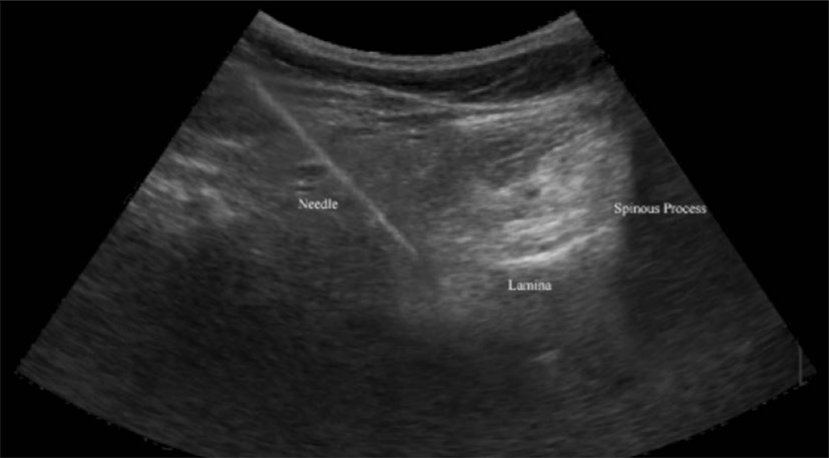
Ultrasound view of needle placement at L4/L5.

The ethical committee of pain research center, neuroscience institute, Tehran University of medical sciences (IR. MEDICINE.1400.878) approved this study, and we explained the associated risks of our procedure with patients, and then informed consent was obtained. We confirm that all experiments were performed in accordance with relevant guidelines and regulations^9^. We assessed 25 eligible patients with the degenerative intervertebral disc at 42 segments with chronic unilateral lumbosacral radicular pain for more than 3 months via the clinical presentation, physical examination, MRI, and nerve conduction velocity (NCV) test from April to July 2021. Fourteen patients had disc hernia, 8 patients had canal stenosis, and 3 cases had spondylolisthesis. Our cases did not show significant improvement of signs and symptoms following conservative therapy such as medication and physiotherapy for more than 6 weeks. The exclusion criteria were infections, previous block within 3 months, taking anti-inflammatory agents, anomalies of the lumbar or sacral spine, previous lumbar spine operation and sever cardiac, pulmonary, liver, and renal dysfunction. The procedure was performed with the patients in the prone position and, in order to decrease lumbar lordosis, a pillow was placed below the lower abdomen. We used an electrical nerve stimulation device with 22 G 100 mm nerve stimulation needles for all cases. The injection areas were sterilized, and a sterile cover was placed on a curved transducer. UNTEB was performed by one experienced pain specialist with 16 years of experience in all our subjects. We placed a curvilinear US probe over the spinous process in a transverse axial scan to visualize the spinous process, lamina, facet joints, and transverse process^10^. Before the procedure, injection areas were infiltrated with 25 G needle containing 1% lidocaine. A nerve stimulation needle 22G was inserted approximately 45° into the skin with the in-plane technique while the path of the needle was seen by ultrasound during the procedure. Electrical current was applied via nerve stimulation needles to evoke the same lumbosacral radicular pain. When nerve stimulator needle reached the lateral side of the lamina or medial to superior articular process, at Z joint location and the same radicular pain evoked by nerve stimulator between 0.5 to 2.5 mA and we ensured that blood or cerebral spinal fluid would not be detected, then 1 ml contrast medium was injected to confirm the correct location of needle in the anterior–posterior and lateral view of fluoroscopy^11^ (Figs. 1, 2, 3, 4). We administered 2 ml of a mixture of 0.25% ropivacaine and dexamethasone (8 mg/ml), one ml of dexamethasone and one ml ropivacaine in each segment during UNTEB in all cases. Before being released from the hospital, all of our individuals were transported to recovery and observed for 2 h. At the outpatient clinic visit 1 day and 1 week following the UNTEB, all patients were assessed for lumbosacral radicular pain using the numerical rating scale (NRS).

### Statistical analysis

The data were analyzed using SPSS version 19 software, and the Wilcoxon signed-rank test was used to compare NRS before and after UNTEB. Analysis of variance was used to compare the demographic characteristics of the patients, and a t-test was used for measurement data. The statistical significance was set at p < 0.05.

### Ethical statement

Written informed consents were obtained from our patient.

## Results

The mean age was 54.8 ± 16.8 years. Sixteen of the patients were male, and nine were female. The procedures were tolerated for all participates. Among 42 foraminal segments, the levels that underwent UNTEB were L3/L4, in 2 cases, L4/L5, in 22 cases and L5/S1, in 18 cases. The same radicular pain was evoked by a nerve stimulator at 0.5, 1, 1.5, 2 and more than 2 mA in 15, 11, 6, and 8 segments. No response was observed in 2 segments. The success ratio of UNTEB in L3/L4 level was 100% (2 of 2), in L4/L5 level was 95.4% (21 of 22), and in L5/S1 level was 100% (18 of 18). There was one failed case at the L4/L5 level during UNTEB. In this case, the needle tip was positioned inappropriately inward to the midline and, therefore, repositioned correctly under US guidance and then confirmed by fluoroscopy. NRS for lumbosacral radicular pain improved from a mean from 7.8 to 2.8 1 day after procedure (p = 0.01) and from a mean from 7.8 to 2.4 1 week after UNTEB (p = 0.01) in our patients. None of our subjects experienced any complications such as numbness of the lower extremities, dizziness, the exacerbation of pain, headache, hemorrhage, infection, and allergic reaction during and after the procedure.

## Discussion

Lumbosacral radicular pain, which commonly occurs throughout life, is caused by spinal canal stenosis, intervertebral disc protrusion or extrusion, and intervertebral disc degeneration. TEB with fluoroscopy guidance was an effective and well-established procedure which was associated with good results in the management of radicular pain^1^. Ultrasonography was broadly used in the assessment and management of the musculoskeletal disorders. It was shown that the ultrasonography was a reliable and accurate technique in evaluating lumbar anatomy and injection of local anesthetic and steroids in these areas for the management of the lumbosacral radicular pain^12^. US-guided TEB was first explained in cadavers in 2005^13^. The first report on the ultrasound-guided selective nerve root in the human was established in 2009^14^, and then the first report of US-guided TEB in the human model was described in 2013^15^. Previous studies showed that lumbar TEB significantly explained better outcomes compared to intralaminar epidural block in management of lumbosacral radicular pain. This could be because the majority of TEB were distributed at the ventral epidural space and provide a high concentration of local anesthetic and steroid at a nociceptive target such as dorsal root ganglions (DRG)^16^. The most challenging part in US-guided TEB is putting the needle exactly in the correct location at the target nerve root and medications spread in epidural space. We use a nerve stimulator to improve the success of needle trajectory and correct target position during US-guided TEB. Our study showed that a nerve stimulator could help us as a supplemental guide for performing a successful US-guided TEB. Previous studies identified the relationship between electrical stimulation and evoked nerve responses based on the needle tip distance^17^. Pain specialists try to determine the exact amplitude at which an evoked response initiates during peripheral nerve block. Initiating at 0.5 mA for selective nerve root block seems to be a very popular amplitude among physicians who apply for peripheral nerve blocks^18^. The positioning of needle closer to the nerve roots needs less electrical current for nerve stimulation, and the response of the nerve root would be more prominent. However, Kim et al. identified that in the TEB, in contrast to a selective nerve root block, the needle has to bypass the nerve root for a successful block^9^. Therefore, it is not necessary that the amplitude at which nerve stimulation is observed have to be very small during the TEB. This result suggests that amplitude up to 2 mA or more could be used during a successful TEB^9^. In our study in 8 segments, the same lumbar radicular pain was evoked in 2 or more than 2 mA. Moreover, in previous studies, authors have explored both axial and parasagittal approaches for US-guided TEB. Recently, some authors used the parasagittal approach because of better vision and easier to perform the block, especially in the upper spine and older patients^6,19,20^. Moreover, most of the authors performed US-guided TEB in in-plane needle trajectory in both approaches because of needle path visualization. One study preferred the parasagittal out-of-plane approach because of the deposition of medication close to the nerve root compared with in-plane technique^10^. Some authors explained possible nerve injury during axial US-guided TEB because the needle tip is not visible in the foramen. Gofeld et al. described a technique that placed the needle tip on the vertebral body instead of the desired space in the foraminal area^21^. All previous studies on US-guided TEB have used another standard method (fluoroscopy or CT) to confirm needle tip position because of the limited vision of the needle tip at the foraminal area due to the bony structure of the lumbar spine^6,22^. We used axial approach and in-plane needle trajectory for UNTEB and confirmed needle tip position by fluoroscopy before injection of medications. The gold standard to assess the success of UNTEB was the relief of symptoms. The success ratio of transforaminal nerve block in our study was 95.4% in L4/L5 and 100% in L3/L4 and L5/S1 levels respectively; therefore, the drug is able to be delivered in transforaminal and epidural spaces well. None of the previous studies identified major complications during and after US-guided TEB^23,24^. We found no complications during and after UNTEB. Our study had some limitations: first, our sample size was small, second, the long-term results were not evaluated, third, the outcomes of the block were assessed only based on pain score, but functional tests of the lumbar spine were not assessed; therefore, these items should be conducted in future researches and fourth, since we examined patients with pain lasting for more than 3 months, it must be stated that this investigation monitored pain only for 1 week. Therefore, no conclusions about long-term outcomes can be made.

We concluded that UNTEB with fluoroscopic validation is a safe, accurate and a success strategy for short-term pain relief of patients with unilateral lumbosacral radicular pain.

## Data Availability

The data that support the findings of this study are available from the corresponding author upon reasonable request.
